# Neuroendocrine and Aggressive-Variant Prostate Cancer

**DOI:** 10.3390/cancers12123792

**Published:** 2020-12-16

**Authors:** Nicholas Spetsieris, Myrto Boukovala, Georgios Patsakis, Ioannis Alafis, Eleni Efstathiou

**Affiliations:** 1 Department of Genitourinary Medical Oncology, University of Texas MD Anderson Cancer Center, Houston, TX 77030, USA; nspetsieris@mdanderson.org (N.S.); m.boukovala@gmail.com (M.B.); ialafis@mdanderson.org (I.A.); 2 Internal Medicine Department, 251 Hellenic Air Force V.A. General Hospital, 11525 Athens, Greece; georgepatsakis@gmail.com

**Keywords:** neuroendocrine prostate cancer, castration resistant prostate cancer, small-cell prostate cancer, aggressive variant prostate cancer, anaplastic prostate cancer

## Abstract

**Simple Summary:**

Prostate cancer may in some cases exhibit microscopic and molecular characteristics of a distinct subtype of disease which is referred to as neuroendocrine differentiation. This entity is rarely found in patients initially diagnosed with metastatic disease and most commonly occurs after treatment of prostate cancer in advanced stages with hormonal agents. This specific presentation of the disease is not effectively targeted by the hormonal therapies used in prostate cancer and exhibits an aggressive clinical course. Interestingly, some tumors may have molecular and clinical characteristics of a neuroendocrine tumor subtype, without however exhibiting the respective histomorphologic features. This aggressive-variant prostate cancer (AVPC) subtype is sensitive to platinum-based chemotherapy, without, however, an impressive long-term response. In this review article we provide an overview of neuroendocrine prostate cancer focusing on the AVPC subtype and we approach current treatment options as well as ongoing research efforts.

**Abstract:**

In prostate cancer, neuroendocrine (NE) differentiation may rarely present de novo or more frequently arises following hormonal therapy in patients with castration-resistant prostate cancer (CRPC). Its distinct phenotype is characterized by an aggressive clinical course, lack of responsiveness to hormonal therapies and poor prognosis. Importantly, a subset of CRPC patients exhibits an aggressive-variant disease with very similar clinical and molecular characteristics to small-cell prostate cancer (SCPC) even though tumors do not have NE differentiation. This aggressive-variant prostate cancer (AVPC) also shares the sensitivity of SCPC to platinum-based chemotherapy albeit with short-lived clinical benefit. As optimal treatment strategies for AVPC remain elusive, currently ongoing research efforts aim to enhance our understanding of the biology of this disease entity and improve treatment outcomes for our patients. This review is an overview of our current knowledge on prostate cancer with NE differentiation and AVPC, with a focus on their clinical characteristics and management, including available as well as experimental therapeutic strategies.

## 1. Introduction

Prostate cancer, the most prevalent cancer in men worldwide, is generally considered a relatively slow-progressing cancer type, ranked fifth in mortality amongst other malignancies [1,2]. This is mainly reflective of the course of prostatic adenocarcinoma, which is by far the most common histologic type of the disease. It is typically androgen-dependent, with androgen deprivation being the backbone of therapy for decades. Despite the almost invariable emergence of castration resistance, most castration-resistant prostatic adenocarcinomas remain dependent on androgen signaling, with novel androgen signaling inhibitors like abiraterone acetate, enzalutamide, apalutamide and darolutamide demonstrating efficacy [3,4,5,6,7,8,9].

Neuroendocrine (NE) tumor differentiation, mainly in the form of pure or mixed small-cell prostate cancer (SCPC), is a histologic subtype with a distinct phenotype, characterized by an aggressive clinical course, lack of responsiveness to hormonal therapies and an overall poor prognosis [10]. While de novo SCPC is rare (<2%), treatment-emergent small-cell differentiation is present in up to 20% of patients with castration-resistant prostate cancer (CRPC) [11,12,13]. Interestingly, there is a subset of CRPC without histologic evidence of NE differentiation that exhibits similar clinical behavior with SCPC, likely indicative of a shared underlying biology [14,15]. This aggressive-variant prostate cancer (AVPC) also shares the responsiveness of SCPC to platinum-based chemotherapy, with the clinical benefit being, however, short-lived and the majority of patients dying within two years from diagnosis [16,17,18,19,20,21]. With the lack of optimal therapies, the mortality rate of these patients has sadly remained unchanged during the last decades, while the incidence of ‘acquired’ NE tumor differentiation and AVPC is rising [22]. Systematic research efforts are imperative in order to enhance our understanding of the biology of this disease entity and improve treatment outcomes for our patients through early detection as well as personalized, effective and safe therapeutic strategies. This review paper presents an overview of our current knowledge on NEPC/AVPC with a focus on their clinical characteristics and management, including available as well as experimental treatments.

## 2. Pathologic Classification of Prostate Cancer with Neuroendocrine Differentiation

NE cells are physiologically scattered within the epithelium of the glands in all anatomic zones of the prostate and are more commonly present in the prostatic gland compared to other organs of the genitourinary tract. By light microscopy, they can be seen in the basal layer between the secretory cells and contain a variety of peptide hormones, such as chromogranin A (CgA), neuron-specific enolase (NSE), serotonin, histamine, calcitonin and neuropeptide Y, vasoactive intestinal peptide, bombesin, and parathyroid hormone-related protein [23,24,25]. NE cells are typically characterized by lack of androgen receptor (AR) and positivity for either synaptophysin, chromogranin, or CD56 in immunohistochemistry [25].

In the last decade, two similar histomorphologic classifications of prostate cancer with NE differentiation have been developed by the World Health Organization (WHO) and the Prostate Cancer Foundation (PCF), in an attempt to systematically describe this heterogeneous prostate cancer subtype [25,26]. (Table 1)

Most prostatic adenocarcinomas contain sparse benign NE cells as a part of the epithelium, but only 5–10% of these tumors will have the characteristic large, multifocal groups of NE cells and fall into the adenocarcinoma with NE differentiation category [27]. Small infiltrating glands with prominent-marginated nucleoli, hyperchromasia, and intraluminal amorphous secretion or blue-tinged mucin as well as isolated tumor cells with eosinophilic granules are frequently present. Immunohistochemical detection of focal areas with at least one NE marker such as CgA, synaptophysin or CD56 may assist in the final diagnostic confirmation of NE differentiation. However, it is controversial whether this subtype is associated with worse oncologic outcomes. Hence, the use of immunohistochemical staining for NE markers in morphologically typical adenocarcinoma of the prostate with the aim to identify NE differentiation is not routinely recommended [25,28].

Well-differentiated NE tumors of the prostate, previously termed carcinoids, morphologically resemble similar tumors of other sites like the gastrointestinal tract and should be distinguished from the clinically aggressive NEPC category, as they tend to have a favorable prognosis [25,29,30,31]. The vast majority of prostatic carcinoids are prostatic adenocarcinomas with “carcinoid-like” morphology, characterized by insular, trabecular, glandular, or mixed architectural pattern and round nuclei with moderately clumped “salt and pepper” chromatin [32]. Pure carcinoids of the prostate are extremely rare. For the diagnosis to be made, the tumor, beyond morphology, must also not be closely associated with concomitant adenocarcinoma of the prostate, it must be positive for NE markers and negative for PSA [25].

De novo SCPC is an exceptionally rare (<2%), but very aggressive and fatal primary cancer [11]. Approximately half of these tumors coexist with typical adenocarcinomas, while the remaining cases present as pure small-cell carcinomas [25]. Histologic characteristics include small, undifferentiated cells with high mitotic activity in the absence of glandular structures, while high nuclear to cytoplasmic ratio, indistinct cell borders and lack of prominent nucleoli are frequent findings. These tumor cells are histologically identical to small-cell lung cancer with a 90% presence of NE markers [33,34]. To distinguish primary SCPC from small-cell carcinomas of other sites, presence of fusion of ETS-related gene with transmembrane protease, serine 2 (TMPRSS2-ERG fusion) is strongly suggestive of a primary prostatic tumor [35,36]. The differentiation of this histologic subtype from adenocarcinoma with NE differentiation and carcinoids is crucial, as it is typically nonresponsive to androgen signaling targeting therapies and requires different treatment. The prognosis is dismal, with a median overall survival of less than one year [16,21].

Large-cell prostatic carcinoma (LCPC) is an extremely rare, aggressive malignancy, with mainly case reports available in the literature [25,37]. It is a high-grade tumor that shows NE differentiation by immunohistochemistry. Morphologically, it consists of cells in large nests with peripheral palisading and geographic necrosis without glandular structures. The cells are characterized by prominent nucleoli, clumpy chromatin, and abundant cytoplasm. Most of the cases described to date have been mixed LCPC with adenocarcinomas [37]. The outcome is poor, with a small case series reporting a mean survival of seven months (range 3–12) after completion of platinum-based chemotherapy following the detection of LCPC [38].

The mixed NE carcinoma-acinar adenocarcinoma included in the PCF meeting classification, is not considered a distinct entity per the 2016 WHO classification [39]. Adenocarcinoma with Paneth cell NE differentiation, which is also included only in the PCF meeting classification, is an entity with incompletely understood clinical significance, with the limited available data pointing to an overall favorable prognosis [40].

A summary of the characteristics of prostate cancer with neuroendocrine differentiation is provided in Table 2 below.

## 3. Aggressive Variants of Castration Resistant Prostate Cancer 

Although small cell/NE differentiation may present de novo in previously untreated patients, it is relatively rare (<2%) [12]. More commonly, NE differentiation develops in castration-resistant patients following androgen deprivation therapy [41]. This phenomenon is relatively common in advanced disease stages, presenting in approximately one fifth of patients with metastatic castration resistant prostate cancer (mCRPC) [13]. Even though the mechanisms of emergence are still under investigation, a recent study points to a model of divergent clonal evolution of CRPC-adenocarcinoma to CRPC with NE differentiation, with adaptation from an AR-driven state to an AR-independent state [42]. An alternative school of thought supports that treatment pressure with androgen signaling inhibitors may enable prostate cancer lineage plasticity and adenocarcinoma trans-differentiation by mechanisms including the acquisition of transcription factors (SOX2,11) and the loss of TP53 and phosphatase and tensin homolog (PTEN) [43,44]. As this phenomenon is limited to a subset of adenocarcinomas, it is crucial to identify baseline features associated with its advent. 

Clinically, treatment-emergent NE/small cell differentiation has been associated with distinct manifestations, including predominantly visceral or lytic bone metastases and bulky tumor masses, frequently in the setting of low PSA level with high-volume tumor burden. Early emergence of castration resistance has also been described [16,19,45,46]. These tumors are typically not responsive to hormonal therapy, while they are sensitive to cytotoxic chemotherapy [47,48]. Responses are however short-lived and overall survival is reduced. Whether outcomes for pure and mixed tumors differ is not definitely answered yet, with some authors suggesting similar clinical behavior [13,16,49], while others report shorter overall survival (OS) for pure SCPC [46]. 

This aggressive variant has been reported more frequently, likely as a result of increasing awareness amongst clinicians and longer survival. Several terminologies have been historically used to describe this CRPC subset, with commonly used terminologies, however, being often ill-defined and having deficiencies [14]. For instance, “NEPC” is an ambiguous term, as it implies the presence of histologic NE differentiation or other NE markers, even though this is not the case in many patients. Also, specific pathology features of NE differentiation are not necessarily associated with an aggressive clinical course (e.g., Paneth cell differentiation). The term “anaplastic” is likely misleading, as this is also an established term designated to pleomorphic cytology by surgical pathologists. The terminology “AR-negative prostate cancer” is considered too limiting, while “therapy-related NE prostate cancer” is being discouraged, as it may drive clinicians to withhold potentially effective hormonal therapies. While the term “AVPC” might be overall less confusing as it does not imply any histologic correlate and is more reflective of the clinical phenotype of this disease, it may potentially be more contaminated. [14]

For the purpose of systematically studying this disease variant, a set of criteria were proposed to define it [19]. CRPC characterized by one or more of the following was determined to be AVPC:

- histologic evidence of SCPC (pure or mixed);
- presence of only visceral metastases;
- predominantly lytic bone lesions;
- bulky (≥5 cm) lymphadenopathy or large (≥5 cm) high-grade (Gleason ≥ 8) tumor mass in prostate/pelvis;
- low PSA at presentation with extensive bone metastatic disease;
- presence of NE markers at histology (CgA and synaptophysin) or serum (CgA and gastrin-releasing peptide) combined with either elevated lactate dehydrogenase (LDH), malignant hypercalcemia or elevated serum carcinoembryonic antigen (CEA);
- progression to CRPC in six months or less after initiation of hormonal therapy.

Of note, the presence of SCPC, either pure or mixed, is considered AVPC regardless of hormonal status [19]. 

In this review, we are going to use the term AVPC according to the principles of the above definition to describe this clinically aggressive-variant disease with or without small-cell histology. AVPC was shown to share the responsiveness of SCPC to platinum based chemotherapy, indicating a likely shared underlying biology [19]. Even though AVPC is a morphologically heterogeneous group of tumors [14,50], it may share molecular characteristics with SCPC. Strikingly, joint alterations in two or more of RB1, TP53 and/or PTEN were shown to correlate with this aggressive clinical phenotype similarly to SCPC, consistent with preclinical models that support the role of combined tumor suppressor alterations in prostate cancer progression and development of resistance to novel hormonal agents [15,43,44,51]. Apart from alterations in tumor suppressors, further features may include AR loss, induction of neuroendocrine/neural as well as mitotic programs, and genomic instability [12,14,15]. 

## 4. Management of AVPC 

### 4.1. Tissue Sampling

In patients with CRPC, biopsy of accessible metastatic lesions should be attempted, as the identification of NE differentiation may impact treatment decisions. A recently reported prospective study in mCRPC described similar clinical characteristics, “typical” of prostatic adenocarcinoma, between patients with and without SCPC, including serum PSA levels and sites of metastasis [13]. This indicates that even patients without “atypical”/aggressive-variant clinical presentation may harbor tumors with NE differentiation and hence diagnostic biopsy of metastatic lesions may be valuable in all mCRPC patients regardless of clinical manifestations. Even though original rapid-autopsy series have reported great histomorphological heterogeneity of metastatic sites within the same patient, suggesting a potentially wide variation of NE features across different metastatic sites [52,53], more recently Kumar et al. analyzed 176 primary or metastatic tumors from 63 men with mCPRC at the time of rapid autopsy and suggested that a single metastasis may provide representative information of the oncogenic driver alterations [54]. 

### 4.2. Standard of Care 

As current knowledge about the optimal treatment of AVPC is incomplete, the guidelines of most medical societies make no specific treatment recommendations for this subset. In CRPC with small-cell histology, cytotoxic chemotherapy has been associated with improved outcome and is generally considered the preferred treatment option [49,55,56,57]. Similarly to small-cell lung cancer, platinum-based chemotherapy regimens are mainly being employed, with cisplatin/etoposide, carboplatin/etoposide, and docetaxel/carboplatin being the regimens recommended by the NCCN [58]. In patients with clinical AVPC (putting aside pure small-cell histology), there is no clear consensus on the optimal first-line therapy, with 58% of the Advanced Prostate Cancer Consensus Conference (APCCC) 2017 voting in favor of standard mCRPC treatment and 42% of platinum-based chemotherapy [59].

The use of chemotherapy regimens for AVPC has been mainly studied in phase 2 studies, with the majority selecting patients based on the presence of elevated serum NE markers, considered to be a systemic indicator of NE differentiation, and only a few employing histomorphological selection criteria [60,61]. Despite an overall observed response to platinum-based chemotherapy regimens in this subset, toxicity is often high and the limiting factor for further testing and routine use of some combinations. In clinical practice, it is vital to consider the therapeutic index (benefit–risk ratio) of every regimen in each individual patient. Table 3 summarizes clinical research experience.

### 4.3. Platinum-Based Regimens with Etoposide

Since the combination of cisplatin with etoposide proved effective in the treatment of small-cell lung cancer, the same regimen was also studied in poorly differentiated NE tumors, exhibiting significant activity and becoming a commonly used therapy for the last decades [63,64,65,66].

In an attempt to improve the efficacy of the traditional cisplatin/etoposide regimen in prostate cancer [57], Papandreou et al. studied the combination of cisplatin/etoposide and doxorubicin in a phase II trial of 38 patients with histologically-confirmed SCPC (67% pure, 33% mixed) [16]. The benefit-risk ratio of the three-drug combination was considered unfavorable in this study and thus the addition of doxorubicin to cisplatin/etoposide was not recommended for clinical practice.

Carboplatin, which is considered to be an acceptable alternative to cisplatin with moderate activity as a single-agent in mCRPC [67], was combined with etoposide in a phase II trial of patients with mCRPC as a second-line therapy after docetaxel [17]. The combination was fairly well tolerated. Median number of cycles received was three and median PFS in the overall study population was 2.1 months. The phase II GETUG P01, examined the same combination, i.e., carboplatin/etoposide, in patients with anaplastic CRPC and visceral metastases or elevated serum CgA and/or NSE [18]. The objective response rate (ORR) was 9% (*n* = 3 PR and *n* = 1 CR) and the toxicity was high, with febrile neutropenia in four patients (7%) and one toxicity-related death, leading the authors to conclude that the benefit-risk-ratio of this combination is not favorable. 

Of note, the dosage and application mode of carboplatin and etoposide differed in both studies, with GETUG P01 employing lower doses of carboplatin (AUC 4 vs. 5), but higher doses of etoposide (100 mg/m^2^/day i.v. for three days vs. 80 mg/m^2^/day i.v. on day 1 and p.o. on days 2 and 3)—a drug known for its myelotoxicity. Also, drug exposure in GETUG P01 was longer with median therapy duration of four cycles (vs. three in Loriot et al.). Furthermore, the GETUG P01-population had an overall lower ECOG performance status (PS), with 12 patients (22%) having an ECOG PS 2 at baseline (vs. 5% in Loriot et al.). This might explain the poorer safety profile of this regimen in GETUG P01 and underlines the importance of a good performance status prior to chemotherapy initiation. 

### 4.4. Platinum-Based Regimens with Taxanes

In men with mCRPC, docetaxel is a standard-of-care option [68,69]. Docetaxel also demonstrated modest activity against small-cell lung cancer in phase II trials and was considered suitable for evaluation in combination regimens [70,71]. Since platinum-based chemotherapy has some activity in the subset of CRPC with NE differentiation, its combination with docetaxel was a reasonable attempt towards improved efficacy in this patient population.

The combination of cisplatin with docetaxel was studied by Culine et al. in a phase 2 study of 41 mCRPC patients with elevated serum NSE and/or CGA [62]. Almost half of the patients experienced a PSA response (i.e., PSA decline ≥50%), and 12 patients (41%) objective partial response. Median OS was 12 months. The safety profile of the combination was poor with 91% experiencing Grade 3–4 neutropenia and one patient dying from sepsis. 

In an effort to improve safety, Aparicio et al. examined the combination of carboplatin/docetaxel in a phase 2 trial of 120 mCRPC patients with clinical AVPC followed by second-line etoposide/cisplatin as salvage therapy [19]. A median of four cycles of carboplatin/docetaxel were administered. PSA response (i.e., PSA decline ≥50%) at course 2 was achieved in 47% of the patients, while objective response of measurable disease in 34%. Median PFS on carboplatin/docetaxel was 5.1 months. Median OS was 16 months. Toxicity was fairly manageable overall, with most common Grade 3 events being infection (*n* = 8) and febrile neutropenia (*n* = 3). Grade 4 events included thrombosis (*n* = 2) and thrombocytopenia (*n* = 1). One toxicity-related death was reported (sepsis during etoposide/cisplatin therapy). 

Building on these results, Corn et al. conducted a phase 2 randomized trial of cabazitaxel vs. cabazitaxel plus carboplatin in patients with mCRPC stratified for the presence of AVPC (ca. 55% per arm) [20]. The platinum-based combination demonstrated improved efficacy, especially in the AVPC subgroup. More specifically, median PFS was improved in the combination arm vs. cabazitaxel alone (7.3 vs. 4.5 months), with prespecified subgroup analysis demonstrating that the platinum-combination favored only those with clinical AVPC (HR 0.58; 95% CI 0.37–0.89). A post hoc analysis for the effect of the molecular AVPC signature, i.e., alterations in two or more of RB1, Tp53 and/or PTEN, demonstrated a similar trend. Median OS was similar between the two arms (HR 0.89, 95% CI 0.63–1.25, *p* = 0.50), with no subgroup analysis available. The combination regimen was tolerated fairly well with a median of six cycles received. 

## 5. Targeted Therapies 

In the emerging era of precision medicine, germline and somatic tumor testing is being strongly recommended in men with high-risk locally advanced and metastatic prostate cancer. Due to the associated prognostic and therapeutic implications, testing for deficiencies in DNA damage response (DDR) as well as DNA mismatch repair (MMR) and resulting microsatellite instability (MSI) should definitely be included in testing [72]. Positive results may be used to guide therapeutic decisions, which may be particularly relevant for patients with AVPC who have an overall poor prognosis and limited alternative treatment options. Germline testing findings are particularly pertinent in preventive strategies for other primary malignancies that may ensue and for family member cancer prevention strategy.

### 5.1. Immunotherapy Options 

MMR gene mutations resulting to phenotypic MSI and/or MMR-deficiency include somatic or germline alterations in MLH1, MSH2, MSH 6, and PMS2 and may be associated with Lynch syndrome, also known as hereditary nonpolyposis colorectal cancer (HNPCC). According to available data, approximately 3–5% of patients with prostate cancer have MSI-high/indeterminate or MMR-deficient tumors and <1% have Lynch syndrome [73,74]. Knowledge of the presence of these molecular alterations is crucial, since they may be targetable with immune checkpoint inhibitors like Pembrolizumab, an antiprogrammed cell death protein 1 (PD-1) antibody, that was approved as a second or later line therapy for MSI-high/MMR-deficient advanced solid tumors in 2017 [75]. In June 2020 FDA approval was also granted to Pembolizumab for the treatment of tumors with a high tumor mutation burden (TMB), namely 10 mutations per megabase and higher assessed by the FoundationOne CDx assay, which was also approved at the time as the companion diagnostic [75,76]. It is noteworthy that Pembrolizumab is the first oncologic drug ever to receive indication based on a molecular, rather than clinical, diagnosis, signifying the beginning of personalized medicine. This approval was based on results of five single-arm trials of Pembrolizumab in patients with MSI-high/MMR-deficient tumors that demonstrated an overall ORR of 40% (59/149) (complete response rate 7%) and a ≥six-month response in 78% of the patients [75,77]. Only two patients with mCRPC were included in these trials, of whom one achieved OR and one stable disease for >nine months. Subsequent prospective and retrospective studies including small numbers of prostate cancer patients with MSI-high/MMR-deficient tumors also reported favorable and often durable responses, although not all patients derived benefit from the treatment [74,78,79,80] Pembrolizumab has an acceptable safety profile, with the most recent data in 258 patients with mCRPC reporting treatment-related AEs in 60% and Grade 3–5 AEs in 15% with the most common treatment- related being fatigue, diarrhea, decreased appetite and immune mediated colitis [81]. The approval of Pembrolizumab for the treatment of high TMB tumors was based on the results of the KEYNOTE- 156 basket study. Results of the study demonstrated that among 790 evaluable patients throughout a 37-month median follow-up period, objective response to Pembrolizumab treatment was observed in 29% of patients with high TMB versus 6% in the non-high TMB cohort [82].

To our knowledge, the exact prevalence of MSI-high/MMR-deficient tumors in patients with AVPC and subsequently the clinical relevance of Pembrolizumab’s indication in this mCRPC subset is yet to be understood. Although Aggarwal et al. reported that the presence of somatic mutations in the MMR genes MLH1, MSH2, MLH3 and MSH6 was almost entirely mutually exclusive (in seven out of eight patients) with the presence of mixed or pure small-cell histology in their mCRPC population [13], others report contradictory results. More specifically, Guedes et al. detected relatively high rates of MSH2 loss in primary tumors with NE differentiation (2/43, 5%), while three out of 32 MSI-high/MMR-deficient patients (9%) in the case series of Abida et al. had NE/small-cell tumor histology [74,83]. Also, several individual cases of patients with histomorphological NE differentiation and presence of MSI/MMR-deficiency have been reported in the literature [78,84]. Of note we now know that microsatellite status may alter to MSI with the advent of genomic alterations over time. Taken together, testing for MSI/MMR-deficiency should be encouraged in patients with AVPC and Pembrolizumab should be offered as a second or later line therapy in these patients as indicated.

Regardless of MSI/MMR status, favorable results from a randomized, double-blind phase III study of patients with small-cell lung cancer treated with the addition of the anti-PDL1 antibody atezolizumab to standard chemotherapy (carboplatin/etoposide) suggest by extrapolation that patients with other NE tumors, may also derive benefit from checkpoint inhibition with or without platinum-based chemotherapy [85]. To answer this question, studies on the use of checkpoint inhibitors alone or in combinations specifically in patients with AVPC are currently under way (discussed below in Section 6.2 and Table 4).

### 5.2. DNA Damage Response (DDR) Pathway 

Patients with metastatic prostate cancer exhibit a relatively high rate of germline mutations in DDR genes, identified in 11.8% of cases unselected for family history. These mutations include alterations in BRCA2, ATM, CHEK2, BRCA1, RAD51D, and PALB2 (in descending frequency order) [86]. Somatic mutations in DDR genes are present in 23% of far advanced mCRPC, with the most commonly mutated gene being BRCA2 in 12.7% [87]. Knowledge of the patients’ germline and somatic DDR status is relevant for therapeutic decisions, as DDR- and especially BRCA2-mutated prostate tumors are responsive to Poly (ADP-ribose) polymerase (PARP) inhibitors and two agents (olaparib, rucaparib) have been recently approved with that indication in mCRPC [88,89]. Response to platinum based therapy is anticipated but has not been tested in a phase III setting.

The prevalence of DDR alterations in AVPC is controversial, as they were almost entirely mutually exclusive with small-cell histology in the series of Aggarwal et al., while others report conflicting results [13,90]. Also, platinum-based chemotherapy is considered the standard first-line treatment option for SCPC regardless of DDR status. Taken together, the relevance of DDR mutations as a therapeutic target in the subset of AVPC remains questionable at this point. Investigation of PARP-inhibitors in AVPC is currently under way (discussed below in Section 6.2 and Table 4).

## 6. Ongoing Research and Future Perspectives 

### 6.1. Neuroendocrine Markers 

There is an urgent need for biomarkers to aid in the early detection of this disease subset, thus hopefully improving outcome by earlier intervention. Noninvasive methods of detection that will potentially spare some patients from invasive biopsy procedures and their associated complications are of particular interest. These include serum NE markers, liquid biopsies and novel imaging techniques. 

Serum NE markers such as NSE and CgA have been reported to be prognostic in patients with mCRPC [61,91,92,93,94,95,96,97,98]. Their role as predictors of response in unselected mCRPC patients is still under investigation, with some reports suggesting such a role [93]. For instance, CgA rise and velocity have been reported to be associated with androgen-independent progression under hormonal therapy [99], while CgA and NSE elevation during the first months of treatment with abiraterone was found to be associated with worse treatment outcome [98]. With regard to chemotherapy, baseline CgA levels and early high CgA rise were shown to be associated with outcome during treatment with taxanes [97,100]. Currently, a prospective cohort study (NCT03017794) is investigating the role of CgA as a predictor of recurrence after primary radiation to the prostate, hypothesizing that radiation-induced NE differentiation is a driver of progression. 

Serum NE markers like NSE and CgA have also demonstrated high negative predictive value in the detection of NE tumor differentiation in patients with androgen-independent metastatic disease, suggesting that diagnostic biopsy aiming at the detection of NE differentiation may be deferred in selected cases [13,45]. However, larger prospective studies are still required to validate these findings and obtain robust thresholds to guide clinical decisions. 

Liquid biopsy has been recently included in the evaluation of patients with advanced prostate cancer with the FDA approval as a companion diagnostic across solid tumors, thus providing a segue to the further development of such assays for more precise disease subtyping [76]. Liquid biopsy techniques are also being investigated as tools for the detection of AVPC. In a prospective study, circulating tumor cells (CTCs) from mCRPC patients including those with NE histology were collected, allowing the investigators to identify a set of morphologic CTC characteristics (low or absent AR expression, low cytokeratin (CK) expression, and smaller size) that may classify tumors with NE differentiation and potentially also AVPC [101]. A previously identified signature consisting of combined alterations in RB1, Tp53, and/or PTEN was applied using circulating tumor (ct)-DNA demonstrating that clinically defined AVPC correlates with the presence of the pre-specified molecular signature in ct-DNA [15,20]. However, the molecular signature in ct-DNA and immunohistochemistry (IHC) did not correlate well. Further studies applying these classifiers in larger cohorts along with molecular analysis of CTCs and ct-DNA are required to further develop liquid biopsies as a diagnostic and/or prognostic tool in clinical practice.

Novel imaging techniques, such as positron emission tomography (PET)/computed tomography (CT) and metabolic magnetic resonance imaging (MRI), may also have the potential to serve as a tool for the noninvasive detection of NE tumor differentiation and are currently under investigation in several studies. In a phase 2 study, gallium Ga 68-DOTATATE PET/CT is being evaluated as a predictive biomarker of NE transdifferentiation in mCRPC (NCT03448458). Triple-tracer PET/CT using 18F-FDG, 68Ga-PSMA and 68Ga-OCTREOTATE is also being studied in mCRPC patients in an observational cohort study (NCT04000776), with coprimary objectives of the detection of NE differentiation and intrapatient intermetastasis polyclonality. MRI with hyperpolarized pyruvate (13C) is currently under investigation in a phase I study (NCT02911467), with the comparison of imaging parameters between adenocarcinoma and tumors with NE differentiation being part of the planned analysis. Results from these diagnostic studies are expected within the next couple of years and will deepen our understanding of the potential role of novel imaging techniques in advanced prostate cancer.

### 6.2. Experimental Therapies 

Since the optimal treatment for AVPC remains elusive, participation in clinical trials is strongly recommended in this patient population [58]. In the currently ongoing trials, patient selection is mostly based on histologic criteria, in contrast to older studies where patients were often selected based on elevated levels of serum NE markers [17,18,62]. Results from these interventional studies are expected to shape future therapeutic strategies in this distinct prostate cancer subset. Ongoing research efforts are discussed below (Table 4).

In the ongoing phase 2 STAMP trial (“Selective Treatment According to Molecular Subtype of Prostate Cancer”—NCT03696186), mCRPC patients will be initially assigned to three groups based on their baseline immunohistochemistry, followed by random assignment to standard or experimental treatment (different in each group). Patients with NE-type tumors will be randomized to androgen deprivation therapy (ADT) + Docetaxel vs. ADT + Docetaxel/Carboplatin, allowing for a systematic evaluation of this commonly used chemotherapy regimen in patients with NE tumors, also in relation to other mCRPC subsets.

Immunotherapy is a novel treatment approach currently attracting increasing scientific interest in the field of prostate cancer. Immune checkpoint inhibition with monoclonal antibodies against cancer immune evasion (PD-L1/2, PD-1, CTLA-4) is currently being studied in combinations or alone in several phase 1/ 2 interventional trials for NEPC and is indicative for the generalized interest for these promising agents. (Table 4) Additionally, newer antigen receptor (CAR) T cell therapies targeting prostate-specific membrane antigen (PSMA) exhibited promising results in mouse models and might point to a new treatment approach against CRPC [102,103]. Future development of CAR-T cells targeting specifically cells with NE differentiation might also be promising. 

Targeted agents against pathways that have been implicated in NE transdifferentiation and/or progression are also being investigated, with partly promising data emerging. Rocalpituzumab is a monoclonal antibody that targets deltalike ligand 3 (DLL3), a protein also expressed in NE CRPC [104]. After showing favorable clinical activity in small-cell lung cancer, it is currently under investigation in other advanced tumors including NEPC (NCT02709889). The cell cycle kinase Aurora-A (AURKA) has been proposed to be involved in the development of NEPC in cooperation with n-myc and AURKA inhibition has been found to suppress NE marker expression both in vivo and in vitro [12,105]. In a recent phase 2 clinical trial with the AURKA-inhibitor alisertib, some patients with n-myc and AURKA overactivity derived significant clinical benefit from targeting this pathway [90]. Enhancer of zeste homolog 2 (EZH2) overexpression has been associated with prostate cancer progression [43,106], with available data pointing to a particular role of EZH2 in the development of NE tumor differentiation [107,108]. EZH2 inhibitors are currently being tested alone (NCT03460977) or in combinations in phase 1/2 trials for the treatment of mCRPC, with, however, most studies not explicitly including patients with NE tumors. Since EZH2 inhibitors have demonstrated a synergistic activity with enzalutamide in the inhibition of cell proliferation by overcoming intrinsic enzalutamide resistance [109], the combination of EZH2 inhibitors with androgen signaling inhibitors is currently under investigation. While the phase 1b/2 ProSTAR study (NCT03480646) will examine the combination of an EZH2 inhibitor with enzalutamide versus abiraterone alone in mCRPC patients with confirmed adenocarcinoma, a similar phase 1b/2 study (NCT04179864) with either abiraterone or enzalutamide in combination with the EZH2 inhibitor Tazemetostat, which has already received accelerated FDA approval for epithelioid sarcoma, will also permit the enrollment of patients with NEPC. 

### 6.3. Treatment Response Monitoring

While the only valid response monitoring approach to date remains imaging, several other markers have been under investigation in the hope to provide clinicians and patients with more sensitive, easily accessible and affordable alternatives. Serum markers like NSE and CgA as well as CEA and LDH have been widely used as surrogates of therapy response, with available data on the ability of NE marker changes to reflect clinical response being contradicting. Papandreou et al. reported parallel decline of serum markers with clinical response in mCRPC patients treated with chemotherapy, with all patients with radiographic response of measurable disease and/or improved or stable bone disease also experiencing a CEA and LDH response (i.e., ≥50% decline) [16]. However, NE response rates do not correspond with the PSA/radiographic response rates in several other studies. For instance, NSE declined at a markedly higher rate compared to the observed PSA/radiographic response rates in the GETUP-P01 trial (31% vs. <10%) [18], while the serum NE marker response rates were lower than the PSA /radiographic response rates in other chemotherapy studies [61,62]. In patients with histologic/clinical NEPC, baseline CTC and trend while on treatment may also be, similarly to the rest of the CRPC population, prognostic [110]. Further data on the value of these and other markers in the monitoring of patients with AVPC are needed to routinely integrate those in clinical practice guidelines.

## 7. Conclusions

AVPC is a disease entity encompassing tumors with or without histologic NE differentiation that present with an aggressive phenotype. These tumors are typically refractory to hormonal therapies and, although they usually respond well to platinum-based chemotherapy regimens by imaging criteria, these responses are short-lived, with these patients having a dismal prognosis overall. Results of currently ongoing preclinical and clinical studies are expected to enhance our understanding of these tumors’ underlying biology and guide our efforts towards the development of personalized medicine through targeted diagnostic and therapeutic approaches. The advent of anti-PD1 treatment in prostate cancer, driven by interrogation of core or liquid biopsy samples for TMB and MSI status, is a promising development in the treatment of this subtype of prostate cancer. The recent approval of a validated companion diagnostics test provides hope towards the direction of a personalized approach of this heterogeneous histologically and clinically disease subtype. Furthermore, agents targeting pathways implicated in the differentiation of prostatic adenocarcinoma to a neuroendocrine subtype are being investigated. Agents currently being studied towards this direction include the DLL3 monoclonal antibody Rocalpituzumab, the AURKA-inhibitor alisertib and the EZH2 inhibitor Tazemetostat. Conclusively, we are gradually entering the era of precision medicine where a one-size fits all approach is considered outdated. Hopefully, we will thus be able to improve the outcomes of patients with this aggressive-variant disease in a clinically meaningful way.

## Figures and Tables

**Table 1:** Classifications of prostate cancer with neuroendocrine (NE) differentiation.

Morphologic Subtype	2016 WHO Classification	PCF Classification
Adenocarcinoma with neuroendocrine differentiation	✔	✔
Well-differentiated neuroendocrine tumor/ carcinoid	✔	✔
Small-cell neuroendocrine carcinoma	✔	✔
Large cell neuroendocrine carcinoma	✔	✔
Adenocarcinoma with Paneth cell neuroendocrine differentiation	_	✔
Mixed neuroendocrine carcinoma–acinar adenocarcinoma	_	✔

PCF = Prostate Cancer Foundation, WHO = World Health Organization.

**Table 2:** Characteristics of prostate cancer with NE differentiation.

Histologic Phenotype
○ Pure small-cell prostate cancer
○ Mixed small-cell prostate cancer
○ Subtypes classified per WHO 2016 and PCF classification
Tumor Markers
○ CgA
○ synaptophysin
○ CD56
○ NSE
Presentation
○ De novo
○ Emerging in castration resistant patients following ADT
Clinical course
○ Aggressive, poor prognosis
○ Treatment-emergent subtype: predominantly lytic/visceral metastases, bulky tumor masses, low PSA
○ Unresponsive to androgen targeted therapies
○ Short-lived response to platinum-based chemotherapy

**Table 3:** Chemotherapy trials in aggressive-variant prostate cancer (AVPC).

Study	Papandreou et al. [16]	Loriot et al. [17]	GETUG P01 [18]	Culine et al. [62]	Aparicio et al. [19]	Corn et al. [20]
**Study design**	Phase 2, single-arm	Phase 2, single-arm	Phase 2, single-arm	Phase 2, single-arm	Phase 2, single-arm	Phase 2, randomized
**Drug combination**	Cisplatin/etoposide + doxorubicin	Carboplatin/etoposide	Carboplatin/etoposide	Cisplatin/docetaxel	Carboplatin/docetaxel (then second-line cisplatin/etoposide)	Carboplatin/cabacitaxel vs. cabacitaxel
**Patient population**	Histologically-confirmed SCPC (pure or mixed)	CRPC after docetaxel with or without elevated NSE/CgA	mCRPC with visceral metastasis or elevated NSE/CgA	mCRPC with elevated NSE/CgA	AVPC (per clinical criteria)	mCRPC stratified by presence of AVPC (per clinical criteria)
***n***	38	40	60	41	121	160
**Efficacy**	36% PSA response 61% OR of measurable disease 84% pain improvement Median PFS 5.8mo Median OS 10.5mo	23% PSA response 2 out of 5 OR of measurable disease 54% pain improvement Median PFS 2.1 mo Median OS 19mo Note *: No association of outcome with NSE/CGA levels	8% PSA response 9% OR of measurable disease No pain evaluation Median PFS 2.9 mo Median OS 9.6 mo	48% PSA response 41% OR of measurable disease 45% pain improvement Median OS 12 mo	47% PSA response (at course 2) 34% OR of measurable disease Median PFS 5.1 mo Median OS 16 mo	62 vs. 41% PSA response 57 vs. 21% OR Median PFS 7.3 vs. 4.5 mo Median OS 18.5 vs. 17.3 mo Note *: PFS and OS improvement with combination greater in AVPC subgroup (clinical and/or molecular)
**Safety—Grade 3–4 AEs >15%**	100% neutropenia 68% infection 66% thrombocytopenia 34% nausea 26% anemia 21% vomiting	38% neutropenia (2% neutropenic fever) 25% anemia	66% neutropenia (7% neutropenic fever) 33% thrombocytopenia 27% anemia	91% neutropenia (17% neutropenic fever) 34% anemia 17% thrombocytopenia 15% fatigue	None	23% anemia 20% fatigue
**Safety—Toxicity-related deaths**	3 (sepsis)	None	1 (febrile neutropenia)	1 (sepsis)	1 (sepsis during second-line etoposide/cisplatin)	1 (thromboembolic event in cabazitaxel arm)

AVPC = aggressive variant prostate cancer, CgA = chromogranin A, mCRPC = metastatic castration resistant prostate cancer, NSE = neuron-specific enolase, OR = objective response, OS = overall survival, PSA = prostate specific antigen, PFS = progression-free survival, SCPC = small-cell prostate cancer. * Results refer to the overall study population (including patients with and without AVPC).

**Table 4:** Ongoing interventional studies in AVPC.

NCT Number	Design	*n*	Patient Population	Interventions	Primary Endpoint(s)	Key Secondary Endpoint(s)	Status
Checkpoint inhibitors							
NCT03582475	Phase 1b, single-arm	30	Locally advanced or metastatic small-cell/NE cancers of urothelium or prostate	Pembrolizumab + platinum-based chemotherapy	DRR, ORR, DOR, OS, PFS	Safety	Recruiting
NCT03910660	Phase 1b/2, single-arm	40	mCRPC with SCPC	Pembrolizumab + Talabostat Mesylate (dipeptidyl peptidase-inhibitor)	Composite response rate	PFS, OS, DOR, safety	Recruiting
NCT02834013	Phase 2, non-randomized	818	Rare tumors, including treatment-emergent SCPC	Ipilimumab +Nivolumab or Nivolumab alone	ORR	PFS, OS, safety	Recruiting
NCT03866382	Phase 2, single-arm	186	Metastatic rare genitourinary tumors, including SCPC	Ipilimumab + Nivolumab + Cabozantinib	ORR	PFS, OS, DOR, safety	Recruiting
NCT03179410	Phase 2, single-arm	18	Metastatic NE-like prostate cancer (per histologic or clinical criteria)	Avelumab	ORR	rPFS, OS, safety	Recruiting
NCT03551782	Phase 1, non-randomized	98	mCRPC, including treatment-emergent SCPC	Cetrelimab + Apalutamide	Safety, PSA response	CTC response	Recruiting
PARP inhibitors							
NCT03263650	Phase 2, randomized	96	AVPC	Cabazitaxel + Carboplatin, followed by olaparib maintenance vs. observation	PFS	ORR, OS, genomic DDR-alterations	Recruiting
Other therapies							
NCT02709889	Phase 1/2, single-arm	200	Patients with delta-like protein 3 (DLL3)- expressing advanced solid tumors, including NEPC	Rovalpituzumab tesirine (anti-DLL3 antibody)	MTD, safety	ORR, DOR, PFS, OS	Terminated
NCT04179864	Phase 1, non-randomize	48	mCRPC, including NEPC	Tazemetostat (EZH2 inhibitor) + abiraterone or enzalutamide	Safety, recommended phase 2 dose	PSA response, CTC conversion	Recruiting
NCT03696186	Phase 2, randomized	300	mCRPC, including a group with histologic NE phenotype	Docetaxel vs. Docetaxel+Carboplatin (in NE group)	OS	PFS, PSA response	Recruiting

AVPC = aggressive variant prostate cancer, CTC = circulating tumor cells, DDR = DNA damage response, DOR= duration of response, DRR = durable response rate, mCRPC = metastatic castration resistant prostate cancer, MTD = maximum tolerated dose, ORR = objective response rate, OS = overall survival, (r)PFS = (radiographic) progression-free survival, SCPC = small-cell prostate cancer.
